# Robustness of Network Controllability with Respect to Node Removals Based on In-Degree and Out-Degree

**DOI:** 10.3390/e25040656

**Published:** 2023-04-14

**Authors:** Fenghua Wang, Robert E. Kooij

**Affiliations:** 1 Faculty of Electrical Engineering, Mathematics and Computer Science, Delft University of Technology, 2628 CD Delft, The Netherlands; 2 Unit ICT, Strategy and Policy, Netherlands Organisation for Applied Scientific Research (TNO), 2595 DA Den Haag, The Netherlands

**Keywords:** controllability, complex networks, node removals

## Abstract

Network controllability and its robustness have been widely studied. However, analytical methods to calculate network controllability with respect to node in- and out-degree targeted removals are currently lacking. This paper develops methods, based on generating functions for the in- and out-degree distributions, to approximate the minimum number of driver nodes needed to control directed networks, during node in- and out-degree targeted removals. By validating the proposed methods on synthetic and real-world networks, we show that our methods work reasonably well. Moreover, when the fraction of the removed nodes is below 10% the analytical results of random removals can also be used to predict the results of targeted node removals.

## 1. Introduction

Network controllability is a crucial area of research that has been explored in various types of networks, including biological networks [1], transportation networks [2], and corruption networks [3]. The controllability of a network refers to the ability to steer the states of its nodes to any desired state in a finite time by manipulating the input to a subset of its nodes. Nodes whose inputs are imposed are named driver nodes. In linear time-invariant systems, Kalman’s controllability rank condition [4] is the classic method of assessing controllability. However, the method has limitations such as computation complexity and the lack of information about the system’s interaction matrix and input matrix. To overcome these limitations, the concept of structural controllability was proposed [5]. Structural controllability is a property of structural linear time-invariant systems with independently free parameters or fixed zero elements in their interaction and input matrices that satisfy the controllability rank condition. Directed networks are structural systems. Liu et al. [6] developed the algorithm and analytical methods to obtain the minimum number of driver nodes in directed networks with the assumption that the directed network has no self-links and a node’s internal state can only be modified upon interaction with neighboring nodes [7]. Throughout this paper, we will adhere to this assumption. Besides structural controllability, Yuan et al. introduced an exact controllability paradigm to determine the minimum number of driver nodes for undirected networks with arbitrary weights by using the maximum multiplicity [8].

In recent years, network structural controllability has gained increasing attention as a tool to measure and enhance network robustness. Robustness is commonly assessed by measuring network performance under various perturbations [9]. One approach is to randomly remove nodes or links and observe the resulting changes in network performance, while another approach involves targeted attack strategies exploiting specific features of network topology such as betweenness, degree, and closeness. Several studies have investigated the effectiveness of different targeted attack strategies on network controllability. For example, degree-based attacks have been found to be more harmful to network controllability than random attacks [10], while betweenness-based attacks are more damaging in most real-world networks [11]. Additionally, attacking bridge links, which results in a disconnected network, has been shown to be an effective way to destroy network controllability [12]. Another approach to targeted attack strategies involves identifying critical nodes and links whose removal increases the number of driver nodes [6]. Protecting critical links can make random link attacks less efficient [13]. Some studies have found that hierarchical attack strategies targeting critical nodes and links first are more efficient than metric-based attack strategies, such as betweenness- or degree-based strategies in interdependent networks [14]. In addition to assessing the robustness of network controllability under perturbations, some studies have focused on enhancing it. For example, increasing the density of nodes with an in-degree and out-degree equal to one or two has been shown to improve network controllability [15]. Adding links to low-degree nodes and creating multi-loop structures have also been found to increase the robustness of network controllability [16]. Furthermore, different redundant design strategies of interdependent networks, such as betweenness-based and degree-based strategies for node backup and high degree first strategy for edge backup, have been investigated to optimize the robustness of network controllability [17].

In addition to qualitative research, quantitative studies have been carried out to explore the robustness of network controllability under different types of perturbations. Lu et al. [11] developed numerical approximations of random and targeted node attacks based on the degree on Erdös-Rényi (ER) networks, which fit well when the fraction of nodes is below 20%. Sun et al. [13] derived closed-form approximations of the minimum number of driver nodes under various types of attacks, including random link attacks, targeted attacks, and random attacks with protection. Chen et al. [18] developed analytical approximations for the minimum number of driver nodes during random link removal using generating functions. Wang et al. [19] later conducted analytical methods based on generating functions to approximate the network controllability during random and targeted node removal based on the total degree of different kinds of networks. In addition to analytical methods, machine learning has been employed to predict network controllability robustness. Dhiman et al. [20] used machine-learning-based approximations to quantify the minimum fraction of driver nodes under random and targeted link attacks, which performed better than the closed-form approximation proposed by Sun et al. [13]. Meanwhile, by utilizing deep learning techniques, Lou et al. have developed a series of works that employ different convolutional neural network (CNN) frameworks, treating the adjacency matrix as a visual representation, to predict network controllability under random node or link attacks, degree-based targeted node or link attacks, and betweenness-based targeted node and link attacks [21,22,23]. Through the use of these models, they have achieved increasingly precise controllability predictions and demonstrated improved scalability. The quantitative studies provide valuable insights into the robustness of network controllability.

As the analytical approximations for targeted node removals based on node in-degree and out-degree are still lacking, in this paper, we aim to utilize the structural controllability framework for directed networks proposed by Liu et al. [6] to make the analytical approximation for those two kinds of targeted node removals. We validate our proposed methods by applying them to three types of synthetic networks and four real-world communication networks.

The remainder of the paper is structured as follows. In Section 2, we introduce the networks used in our study. Section 3 presents the analytical results of network controllability under the two classes of targeted attacks. Finally, we conclude and discuss the implications of our findings in Section 4.

## 2. Network Data

To validate the theoretical results presented in the following sections, we will utilize three categories of synthetic networks as well as several real-world networks. In this section, we provide specific information regarding the utilized networks.

### 2.1. Directed Synthetic Networks

We choose three types of synthetic networks: Erdös-Rényi (ER) networks, Swarm Signaling networks (SSNs) and Scale-free networks (SFs).

We generate a directed ER network with *N* nodes, whereby a directed link is placed between every pair of nodes with a given probability of $p_{ER}$. The average number of links is governed by the equation, $L=N\left(N-1\right)p_{ER}$. This study has employed two kinds of ER networks with N=50 and N=100, and $p_{ER}=0.07$ and $p_{ER}=0.04$, respectively.

The topology of Swarm Signaling Networks (SSNs), proposed in [24], is characterized by a regular out-degree and an in-degree distribution that follows a Poisson distribution. Two parameters must be specified to generate SSNs: the number of nodes, *N*, and the out-degree value, *k*. Each node in the network randomly creates *k* outgoing links to other nodes. Two kinds of SSNs are chosen, with $N=10^{4}$ and average out-degree values of k=2 and k=5, respectively.

Scale-free networks (SFs) are a class of complex networks whose both in-degree and out-degree distributions exhibit a power-law distribution. In this paper, we generate two SFs using the Barabási–Albert model, which is a preferential attachment mechanism that generates networks with a power-law degree distribution with an exponent γ=3 [25]. Specifically, we generate SFs in two stages. In the first stage, we generate a Barabási–Albert graph with *N* nodes, where the initial state is a star with m+1 nodes. At each step, a node with *m* edges is preferentially attached to existing nodes with high degrees until the total number of nodes reaches *N*. In the second stage, we randomly assign directions to each link in the generated graph. The resulting SFs have in-degree and out-degree distributions that follow a power-law distribution with an exponent γ=3. We set m=5 and m=10 for both SFs with $N=10^{5}$ nodes, resulting in minimum in-degree and out-degree values *a* of 5 and 10, respectively, which are approximated by the integers that make the ceiling of the average value of the power-law distribution equal to *m*.

### 2.2. Real-World Networks

In this study, we employed real-world communication networks obtained from the Topology Zoo dataset [26]. To convert these networks from undirected to directed, we utilized the source and targeted node attributes [13]. Table 1 demonstrates the basic properties of the networks used in this study, including the number of nodes *N*, the number of links *L*, and the average total degree <k>. The total degree of a node is the sum of its in-degree and out-degree. Since the average in-degree equals the average out-degree, the average total degree is twice the average in-degree (and out-degree).

## 3. Network Controllability

Consider a linear, time-invariant networked system of *N* nodes, where each node’s state is governed by $\dot{x}\left(t\right)=Ax\left(t\right)+Bu\left(t\right)$, with $x\left(t\right)={\left(x_{1}\left(t\right),x_{2}\left(t\right),\ldots ,x_{n}\left(t\right)\right)}^{T}$ being the N×1 state vector. The N×N matrix *A* represents the interactions among the network components, and the N×M matrix *B* specifies which nodes are under the direct control of the M×1 control input vector $u\left(t\right)={\left(u_{1}\left(t\right),u_{2}\left(t\right),\ldots ,u_{m}\left(t\right)\right)}^{T}$.

A linear, time-invariant networked system is controllable if it can reach any desired state within a finite time by applying external inputs. The Kalman rank criterion requires that the rank of the controllability matrix $\left[B,AB,A^{2}B,\ldots ,A^{n-1}B\right]$ equals *N* for the system to be fully controllable. Liu et al. introduced the maximum matching method and the minimum inputs theorem to determine the minimum number of driver nodes required to ensure network structural controllability [6]. The number of driver nodes, $N_{D}$, can be obtained by mapping a directed network into a bipartite network [13], obtaining a maximum matching edge set using the maximum matching algorithm [27], and then calculating $N_{D}=\min\left\{1,N-N_{m}\right\}$, where $N_{m}$ is the number of directed edges in the maximum matching set without sharing the same source or end nodes.

## 4. In-Degree and Out-Degree Node Attacks

Centrality analysis is an essential research area in studying network robustness [28]. Nodes with a high degree are known to have a substantial impact on network functioning and are more susceptible to targeted attacks. In this study, our objective is to investigate an analytical approximation of network controllability during targeted node removal based on two types of degrees: in-degree and out-degree.

Assuming that the probability of node attack is proportional to some power of its in-degree and out-degree, we can express the probability of removing node *i* based on its in-degree $k_{in\_i}$ as $p_{in\_i}$ and based on its out-degree $k_{out\_i}$ as $p_{out\_i}$. The formula for calculating these probabilities is given as follows:

(1) $$\begin{array}{cc} p_{in\_i} & =\frac{k_{in\_i}^{\alpha }}{\sum_{j\in N}k_{in\_j}^{\alpha }},\\ p_{out\_i} & =\frac{k_{out\_i}^{\alpha }}{\sum_{j\in N}k_{out\_j}^{\alpha }}. \end{array}$$

In the node removal process, after some nodes are removed, we recalculate the removal probabilities for the remaining nodes using Equation (1). We then select nodes to remove based on the recalculated probabilities until all nodes are removed.

When α=0, the aforementioned equations become

(2) $$\begin{array}{cc} p_{in\_i} & =\frac{1}{N},\\ p_{out\_i} & =\frac{1}{N}, \end{array}$$

which indicates that each node has an equal probability of being removed, resulting in a random removal strategy. On the other hand, for α>0, nodes with higher degrees have a greater likelihood of being removed, while for α<0, nodes with lower degrees are more likely to be removed.

In this study, we investigate the impact of degree-based node removal strategies on network robustness. To this end, we focus on α>0, as higher-degree nodes are commonly targeted for attack in real-world scenarios. Specifically, we consider two values of α, namely α=1 and α=10, to evaluate the impact of removing nodes proportional to their degree and removing high-degree nodes more aggressively, respectively. By using Equation (1), we obtain the probabilities of the node being removed based on in-degree and out-degree when α=1 as follows:

(3) $$\begin{array}{cc} p_{in\_i} & =\frac{k_{in\_i}}{\sum_{j\in N}k_{in\_j}},\\ p_{out\_i} & =\frac{k_{out\_i}}{\sum_{j\in N}k_{out\_j}}.\; \end{array}$$

Analogously, the node removal probabilities based on in-degree or out-degree with α=10 can be calculated by

(4) $$\begin{array}{cc} p_{in\_i} & =\frac{k_{in\_i}^{10}}{\sum_{j\in N}k_{in\_j}^{10}},\\ p_{out\_i} & =\frac{k_{out\_i}^{10}}{\sum_{j\in N}k_{out\_j}^{10}}. \end{array}$$

Our results show that, for α=10, the removal of high-degree nodes does not lead to a significant reduction in network robustness in the beginning stage. For several networks, there are no significant differences between the results with α=1 and α=100. Interestingly, we observe that increasing the value of α to 100 does not result in further performance gains, as the performance of attacks with α=100 is similar to that of attacks with α=10. Additional details on these findings can be found in Appendix A. Furthermore, we find that when α=1, the removal strategies based on in-degree or out-degree can be more detrimental to certain networks than node removal based on the total degree. However, for some other networks, the harmful effects of these strategies are comparable. The results are presented in Appendix B.

## 5. Minimum Fraction of the Number of Driver Nodes under Targeted Node Attacks

### 5.1. Analytical Approximation

The analytical approximation for targeted node removal based on in- and out-degrees with different α is derived from the analytical approximation of random node removal. As such, we begin by introducing the methodology for approximating the minimum fraction of driver nodes under random removal, and then introduce the analytical methods of the cases: α=1 and α=10.

#### 5.1.1. Case: α=0

To predict the minimum fraction of driver nodes under random removal, α=0, by using the analytical method based on generating function of degrees, we first employ the framework proposed by Liu et al. [6]. Given a directed network G(N,L) with *N* nodes and *L* links, we can determine the minimum fraction of driver nodes using the generating function of the in- and out-degree distributions, denoted by $G_{in}\left(x\right)$ and $G_{out}\left(x\right)$, respectively, as well as the excess in- and out-degree distributions, denoted by $H_{in}\left(x\right)$ and $H_{out}\left(x\right)$, respectively. These generating functions can be defined as follows:

(5) $$\begin{array}{cc} G_{in}\left(x\right) & =\sum_{k=0}^{\infty }P_{in}\left(k_{in}\right)x^{k_{in}},\\ G_{out}\left(x\right) & =\sum_{k=0}^{\infty }P_{out}\left(k_{out}\right)x^{k_{out}},\\ H_{in}\left(x\right) & =\frac{\sum_{k=1}^{\infty }k_{in}P_{in}\left(k_{in}\right)x^{k_{in}-1}}{<k_{in}>}=\frac{G_{in}^{\prime }\left(x\right)}{G_{in}^{\prime }\left(1\right)},\\ H_{out}\left(x\right) & =\frac{\sum_{k=1}^{\infty }k_{out}P_{out}\left(k_{out}\right)x^{k_{out}-1}}{<k_{out}>}=\frac{G_{out}^{\prime }\left(x\right)}{G_{out}^{\prime }\left(1\right)}, \end{array}\;$$

where $k_{in}$ and $k_{out}$ represent in-degree and out-degree, respectively, while $P_{in}\left(.\right)$ and $P_{out}\left(.\right)$ are in- and out-degree probability distributions, respectively. Then, the minimum fraction of driver nodes can be obtained by

(6) $$\begin{array}{cc} n_{d} & =\frac{1}{2}\{G_{in}\left(\omega_{2}\right)+G_{in}\left(1-\omega_{1}\right)-2+G_{out}\left(\hat{\omega_{2}}\right)+G_{out}\left(1-\hat{\omega_{1}}\right)\\ \; & +k[\hat{\omega_{1}}\left(1-\omega_{2}\right)+\omega_{1}\left(1-\hat{\omega_{2}}\right)]\}, \end{array}$$

where $\omega_{1}$, $\omega_{2}$, ${\hat{\omega }}_{1}$ and ${\hat{\omega }}_{2}$ satisfy

(7) $$\begin{array}{cc} \omega_{1} & =H_{out}\left({\hat{\omega }}_{2}\right),\\ \omega_{2} & =1-H_{out}\left(1-{\hat{\omega }}_{1}\right),\\ {\hat{\omega }}_{1} & =H_{in}\left(\omega_{2}\right),\\ {\hat{\omega }}_{2} & =1-H_{in}\left(1-\omega_{1}\right), \end{array}$$

and *k* denotes half of the average degree equal to the average in-degree and the average out-degree, $k=\frac{1}{2}<k>=<k_{in}>=<k_{out}>$. We aim to determine the minimum fraction of driver nodes $n_{D}$ needed to control the remaining part of the network after removing a fraction *p* of nodes. To this end, we partition the network into two sets: a set containing $N_{D}$ driver nodes that can control the rest of the network and a set of $N_{r}$ removed nodes. We assume that each removed node requires the control of an individual driver node. Then, we define the fraction of driver nodes $n_{D}$ as $n_{D}=\frac{N_{D}+N_{r}}{N}$. After removing a fraction *p* of nodes from the network, we can obtain the following expression for the minimum fraction of driver nodes $n_{D}$,

(8) $$n_{D}=\frac{n_{d}\left(1-p\right)N+pN}{N}=n_{d}\left(1-p\right)+p.$$

We adopt the method proposed by Shao et al. [29] to adjust the generating functions of in- and out-degree and the excess in- and out-degree after randomly removing a fraction *p* of nodes from the network. According to this method, the generating function after random removal can be obtained by applying an adjusted augmentation $\bar{x}=p+\left(1-p\right)x$ to the original generating functions. Hence, the generating functions of the in- and out-degree and the excess the in- and out-degree after removing a fraction *p* of nodes can be expressed as follows:

(9) $$\begin{array}{cc} {\bar{G}}_{in}\left(x\right) & =G_{in}\left(p+\left(1-p\right)x\right),\\ {\bar{G}}_{out}\left(x\right) & =G_{out}\left(p+\left(1-p\right)x\right),\\ {\bar{H}}_{in}\left(x\right) & =\frac{{\bar{G}}_{in}^{\prime }\left(x\right)}{{\bar{G}}_{in}^{\prime }\left(1\right)},\\ {\bar{H}}_{out}\left(x\right) & =\frac{{\bar{G}}_{out}^{\prime }\left(x\right)}{{\bar{G}}_{out}^{\prime }\left(1\right)}. \end{array}\;$$

Next, we use Equations (6) and (8) to obtain the fraction of the minimum number of nodes $n_{D}$ after removing a fraction *p* of nodes,

(10) $$\begin{array}{cc} n_{D} & =\frac{1}{2}\left(1-p\right)\{{\bar{G}}_{in}\left(\omega_{2}\right)+{\bar{G}}_{in}\left(1-\omega_{1}\right)-2+{\bar{G}}_{out}\left(\hat{\omega_{2}}\right)+{\bar{G}}_{out}\left(1-\hat{\omega_{1}}\right)\\ \; & +k\left(1-p\right)[\hat{\omega_{1}}\left(1-\omega_{2}\right)+\omega_{1}\left(1-\hat{\omega_{2}}\right)]\}+p, \end{array}\;$$

where $\omega_{1}$, $\omega_{2}$, ${\hat{\omega }}_{1}$ and ${\hat{\omega }}_{2}$ satisfy

(11) $$\begin{array}{cc} \omega_{1} & ={\bar{H}}_{out}\left({\hat{\omega }}_{2}\right),\\ \omega_{2} & =1-{\bar{H}}_{out}\left(1-{\hat{\omega }}_{1}\right),\\ {\hat{\omega }}_{1} & ={\bar{H}}_{in}\left(\omega_{2}\right),\\ {\hat{\omega }}_{2} & =1-{\bar{H}}_{in}\left(1-\omega_{1}\right). \end{array}$$

#### 5.1.2. Case: α=1

**In-degree:**  In undirected networks, after a fraction *p* of nodes have been removed based on their degree; specifically, the probability of a node removal is proportional to some power of its degree, see Equation (1); the generating function of the degree distribution, G(x), transforms into function $\bar{G}\left(x\right)$, which is as follows [28]:
  (12) $$\bar{G}\left(x\right)=\frac{1}{1-p}\sum_{k=0}^{\infty }p_{k}f^{k^{\alpha }}{\left(1+\frac{fG_{\alpha }^{\prime }\left(f\right)}{<k>}\left(x-1\right)\right)}^{k},$$
  where $f\equiv G_{\alpha }^{-1}\left(1-p\right)$, $G_{\alpha }\left(x\right)\equiv \sum_{k}p_{k}x^{k^{\alpha }}$ and <k> is the average degree of the initial network.
  We investigate the extension of prior conclusions to directed networks while removing nodes based on their in-degree. We assume that a node’s in-degree and out-degree are independent and uncorrelated, such that removing a fraction *p* of nodes based on their in-degree results in the generating function of the in-degree distribution described by Equation (12). Furthermore, the generating function of the out-degree distribution is given by ${\bar{G}}_{out}\left(x\right)=G_{out}\left(p+\left(1-p\right)x\right)$ following the equation of random node removals. So, if we remove nodes based on in-degree, function ${\bar{G}}_{in}\left(x\right)$ and function ${\bar{G}}_{in}\left(x\right)$ satisfy
  (13) $$\begin{array}{cc} {\bar{G}}_{in}\left(x\right) & =\frac{1}{1-p}\sum_{k_{in}=0}^{\infty }p_{k_{in}}f^{k_{in}}{\left(1+\frac{fG_{1}^{\prime }\left(f\right)}{<k_{in}>}\left(x-1\right)\right)}^{k_{in}},\\ {\bar{G}}_{out}\left(x\right) & =G_{out}\left(p+\left(1-p\right)x\right). \end{array}$$
  Then, we can obtain the analytical approximation of the minimum fraction of driver nodes under node removals based on in-degree using Equation (10).
**Out-degree:**  Analogously, if we remove a fraction *p* of nodes based on their out-degree, we maintain the assumption that the generating function of the out-degree distribution is described by Equation (12). Additionally, the generating function of the in-degree distribution can be expressed as ${\bar{G}}_{in}\left(x\right)=G_{in}\left(p+\left(1-p\right)x\right)$. Therefore, we have function ${\bar{G}}_{in}\left(x\right)$ and function ${\bar{G}}_{in}\left(x\right)$ as follows:
  (14) $$\begin{array}{cc} {\bar{G}}_{in}\left(x\right) & =G_{in}\left(p+\left(1-p\right)x\right),\\ {\bar{G}}_{out}\left(x\right) & =\frac{1}{1-p}\sum_{k_{out}=0}^{\infty }p_{k_{out}}f^{k_{out}}{\left(1+\frac{fG_{1}^{\prime }\left(f\right)}{<k_{out}>}\left(x-1\right)\right)}^{k_{out}}. \end{array}$$
  Furthermore, utilizing Equation (10), we can derive an analytical approximation of the minimum fraction of driver nodes when nodes are removed based on out-degree.

#### 5.1.3. Case: α=10

When α=10, we encounter difficulties in obtaining a numerical solution for $f\equiv G_{\alpha }^{-1}\left(1-p\right)$, where $G_{\alpha }\left(x\right)\equiv \sum_{k}p_{k}x^{k^{\alpha }}$. Consequently, it becomes challenging to determine the evolution of the generating functions for in-degree and out-degree distributions during the node removal process. To address this challenge, we propose a heuristic approach whereby we map the targeted node removal process based on in-degree or out-degree into a random node attack process.

Specifically, for node removals based on in-degree with α=10, where a fraction of *p* nodes are to be removed, we map this process to the removal of $\bar{p}$ nodes in the in-degree distribution, while maintaining the fraction of nodes in the out-degree distribution at *p*. Similarly, for node removals based on out-degree with α=10, we map the process to the random removal of a fraction of $\bar{p}$ nodes in the out-degree distribution, as well as a fraction of *p* nodes in the in-degree distribution.

**In-degree:**  In order to estimate the corresponding $\bar{p}$ of a given fraction *p* under node removals based on in-degree with α=10, we adopt the assumption that nodes are removed in descending order of in-degree. Specifically, we first sort the nodes according to their in-degree and then remove nodes starting from the node with the highest in-degree until the targeted fraction *p* is reached.
  Next, we calculate the total in-degree of all the removed nodes by utilizing the original in-degree distribution and the targeted removal fraction *p*. The effective fraction $\bar{p}$ is then obtained by normalizing the total in-degree of all removed nodes with respect to the total in-degree of all nodes in the initial network. This can be calculated as follows:
  (15) $${\bar{p}}_{in}=\frac{\sum_{k_{in}=k_{in_{max}}}^{k_{in}={\bar{k}}_{in}}p_{k_{in}}Nk_{in}}{N<k_{in}>}=\frac{\sum_{k_{in}=k_{in_{max}}}^{k_{in}={\bar{k}}_{in}}p_{k_{in}}k_{in}}{<k_{in}>},\;$$
  where the largest in-degree value is denoted as $k_{in_{max}}$, the probability of removed nodes with degree $k_{in}$ is denoted as $p_{k_{in}}$ and degree ${\bar{k}}_{in}$ satisfies $\sum_{k_{in}=k_{in_{max}}}^{k_{in}={\bar{k}}_{in}}p_{k_{in}}=p$. It is worth mentioning that except for removed probability $p_{{\bar{k}}_{in}}$, other probability $p_{k_{in}}$ is equal to probability $P_{in}\left(k_{in}\right)$ in the generating function. Then, we can use effective proportion ${\bar{p}}_{in}$ for the approximation of the minimum fraction of driver nodes as follows:
  (16) $$\begin{array}{cc} {\bar{G}}_{in}\left(x\right) & =G_{in}\left({\bar{p}}_{in}+\left(1-{\bar{p}}_{in}\right)x\right),\\ {\bar{G}}_{out}\left(x\right) & =G_{out}\left(p+\left(1-p\right)x\right),\\ n_{D} & =\frac{1}{2}\{{\bar{G}}_{in}\left(\omega_{2}\right)+{\bar{G}}_{in}\left(1-\omega_{1}\right)-2+{\bar{G}}_{out}\left(\hat{\omega_{2}}\right)+{\bar{G}}_{out}\left(1-\hat{\omega_{1}}\right)\\ \; & +k\left(1-\frac{p+{\bar{p}}_{in}}{2}\right)\left[\hat{\omega_{1}}\left(1-\omega_{2}\right)+\omega_{1}\left(1-\hat{\omega_{2}}\right)\right]\}\left(1-\frac{p+{\bar{p}}_{in}}{2}\right)+\frac{p+{\bar{p}}_{in}}{2}, \end{array}\;$$
  where $\omega_{1},\omega_{2},\bar{\omega_{1}}$ and $\bar{\omega_{2}}$ satisfy Equation (11).
**Out-degree:**  Analogously, for targeted node removal based on out-degree with α=10, the calculation of fraction ${\bar{p}}_{out}$ follows the same assumption: nodes are removed from the node with the highest out-degree to the node with the lowest out-degree until the removed fraction of nodes reaches *p*. The effective fraction ${\bar{p}}_{out}$ is the total out-degree of removed nodes normalized by the total out-degree in the original network, which can be calculated by
  (17) $${\bar{p}}_{out}=\frac{\sum_{k_{out}=k_{out_{max}}}^{k_{out}={\bar{k}}_{out}}p_{k_{out}}Nk_{out}}{N<k_{out}>}=\frac{\sum_{k_{out}=k_{out_{max}}}^{k_{out}={\bar{k}}_{out}}p_{k_{out}}k_{out}}{<k_{out}>},$$
  where the largest degree value is denoted as $k_{out_{max}}$, and the probability of removed nodes with out-degree $k_{out}$ as $p_{k_{out}}$. To achieve the targeted removal fraction *p*, we find the minimum out-degree value ${\bar{k}}_{out}$ satisfying $\sum_{k_{out}=k_{out_{max}}}^{k_{out}={\bar{k}}_{out}}p_{k_{out}}=p$. For all out-degree values except for ${\bar{k}}_{out}$, their corresponding probabilities $p_{k_{out}}$ are equal to the probabilities $P_{out}\left(k_{out}\right)$ in the generating function. Then, we use ${\bar{p}}_{out}$, the effective proportion of removed nodes based on out-degree, to estimate the minimum number of driver nodes, which is given by the following expression:
  (18) $$\begin{array}{cc} {\bar{G}}_{in}\left(x\right) & =G_{in}\left(p+\left(1-p\right)x\right),\\ {\bar{G}}_{out}\left(x\right) & =G_{out}\left({\bar{p}}_{out}+\left(1-{\bar{p}}_{out}\right)x\right),\\ n_{d} & =\frac{1}{2}\{{\bar{G}}_{in}\left(\omega_{2}\right)+{\bar{G}}_{in}\left(1-\omega_{1}\right)-2+{\bar{G}}_{out}\left(\hat{\omega_{2}}\right)+{\bar{G}}_{out}\left(1-\hat{\omega_{1}}\right)\\ \; & +k\left(1-\frac{p+{\bar{p}}_{out}}{2}\right)\left[\hat{\omega_{1}}\left(1-\omega_{2}\right)+\omega_{1}\left(1-\hat{\omega_{2}}\right)\right]\}\left(1-\frac{p+{\bar{p}}_{out}}{2}\right)+\frac{p+{\bar{p}}_{out}}{2}, \end{array}\;$$
  where $\omega_{1},\omega_{2},\bar{\omega_{1}}$ and $\bar{\omega_{2}}$ satisfy Equation (11).

### 5.2. Results for Targeted Node Attacks

#### 5.2.1. Case: α=1

We ran simulations on various networks, as described in Section 2. We carried out 10,000 realizations for all networks to ensure sufficient statistical power. For ER and real-world networks, which have a relatively small number of nodes, one node was removed at each step until all nodes had been removed during each realization. Then, a recalculation of the minimum fraction of driver nodes was conducted by using the algorithm. On the other hand, due to the large number of nodes in SSNs and SFs, 1% of nodes were removed at each step until all nodes had been removed during each realization. Subsequently, the minimum fraction of driver nodes was recalculated based on the modified network structure. The average value of results obtained from the 10,000 realizations was taken as the final simulation output.

**Figure 1:**
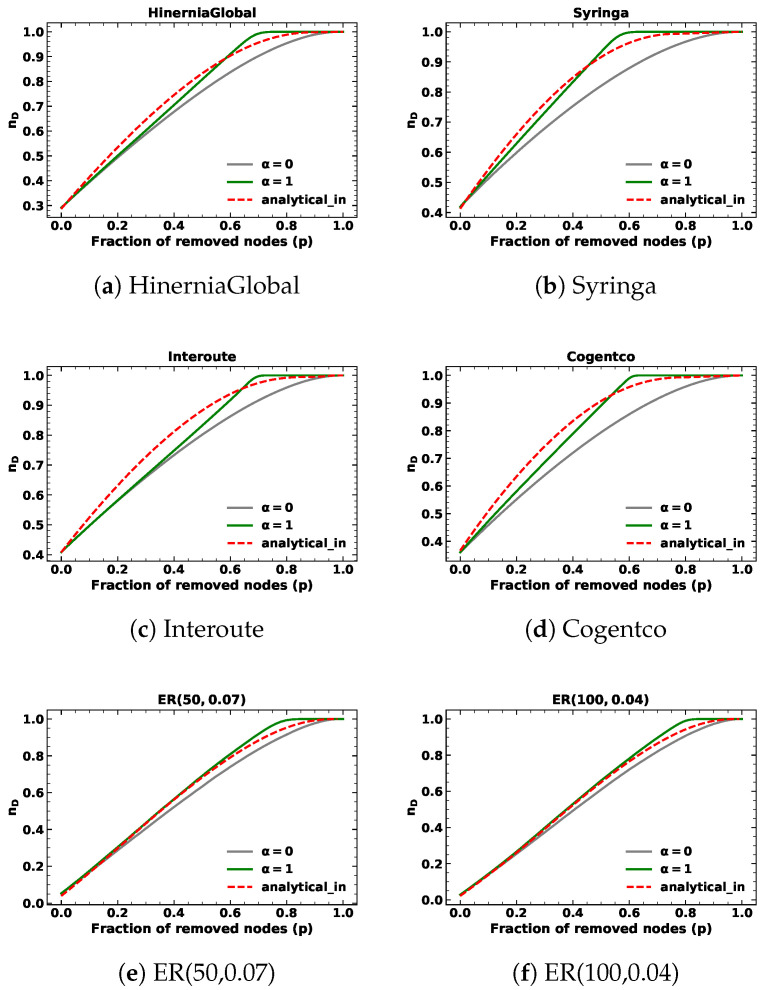

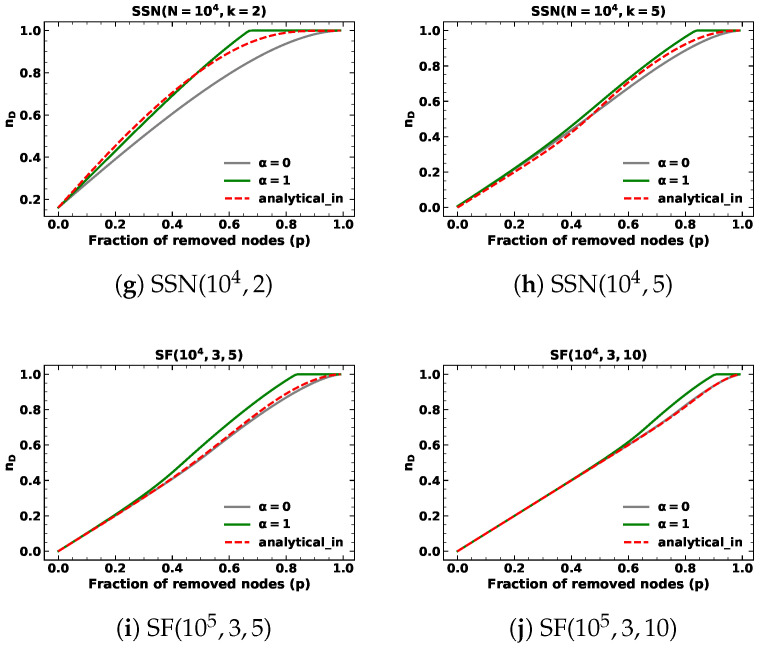
The minimum fraction of driver nodes $n_{D}$ during targeted node removal based on in-degree with α=1 for different kinds of networks.

**Figure 2:**
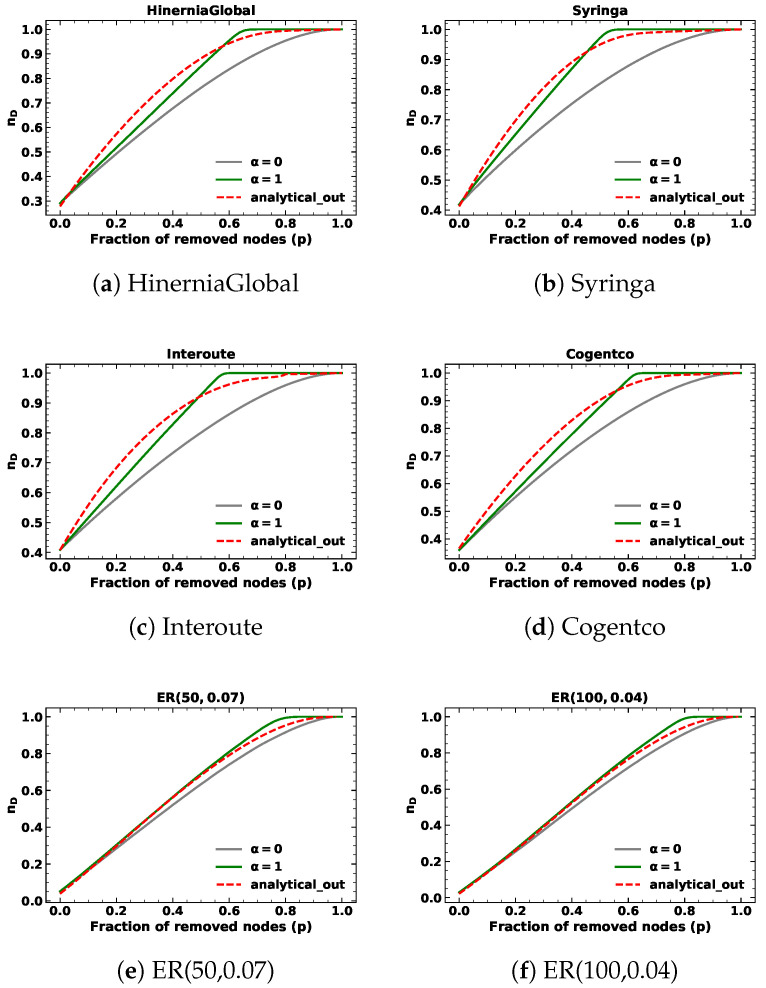

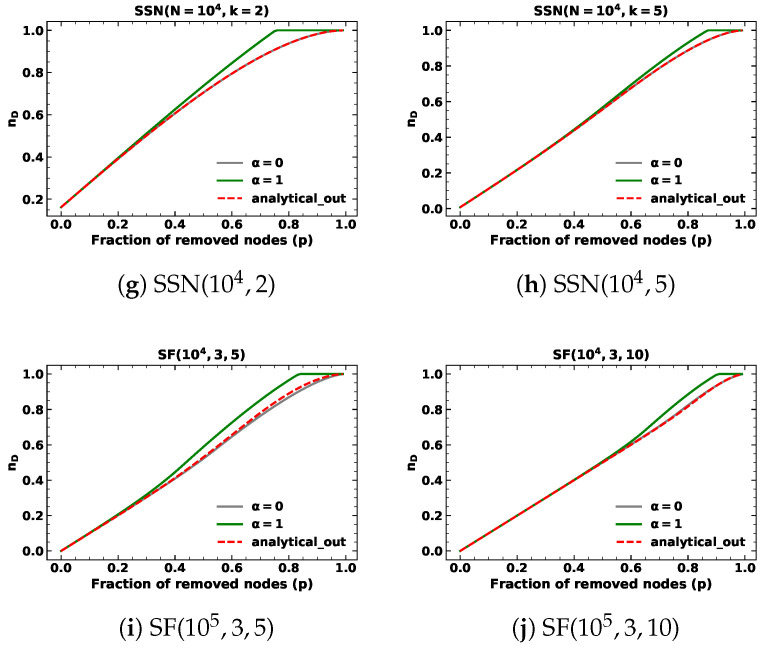
The minimum fraction of driver nodes $n_{D}$ during targeted node removal based on out-degree with α=1 for different kinds of networks.

We present the results of targeted node removal based on in-degree and out-degree with α=1, as depicted in Figure 1 and Figure 2. The simulation results are shown in green lines, whereas the analytical results are in red. The results of random node removal are also presented in gray lines for comparison. We observe that the analytical results serve as a closed-form approximation of the minimum fraction of driver nodes ($n_{D}$), as a discrepancy exists between the predicted and simulation values during the targeted node removal process based on in-degree or out-degree. In the case of ER networks and SFs, the in-degree and out-degree distributions are identical. Consequently, the predicted values of targeted removal based on in-degree and out-degree are also the same. For SSNs, the out-degree of nodes is fixed. Therefore, the lines of analytical results of targeted node removal based on out-degree with α=1 in SSNs overlap with the lines of random node removal. We find that for SFs and SSNs, the simulation results of targeted removal based on in-degree and out-degree are slightly different from the simulation results of random removals. Thus, even though the analytical results of SFs differ slightly from the simulation results of random removals and the analytical results of SSNs are the same as the random removal results, they closely approach the simulation results of targeted removals.

When the removed fraction *p* is small, the simulation results of targeted removals based on in-degree and out-degree are close to those of random removals. We verified this by calculating the Root Mean Square Error (RMSE) between the simulation results of targeted removals based on in-degree and out-degree and analytical results of randomly removing nodes below 10%, as shown in Table 2. Moreover, we calculated the RMSE between the simulation and analytical results of targeted node removals based on in-degree and out-degree below 10%, as shown in Table 3. The results indicated that both methods provide a good approximation of the simulation results, as the values in both tables for targeted node removals based on in-degree and out-degree with α=1 are reasonably small.

#### 5.2.2. Case: α=10

**Figure 3:**
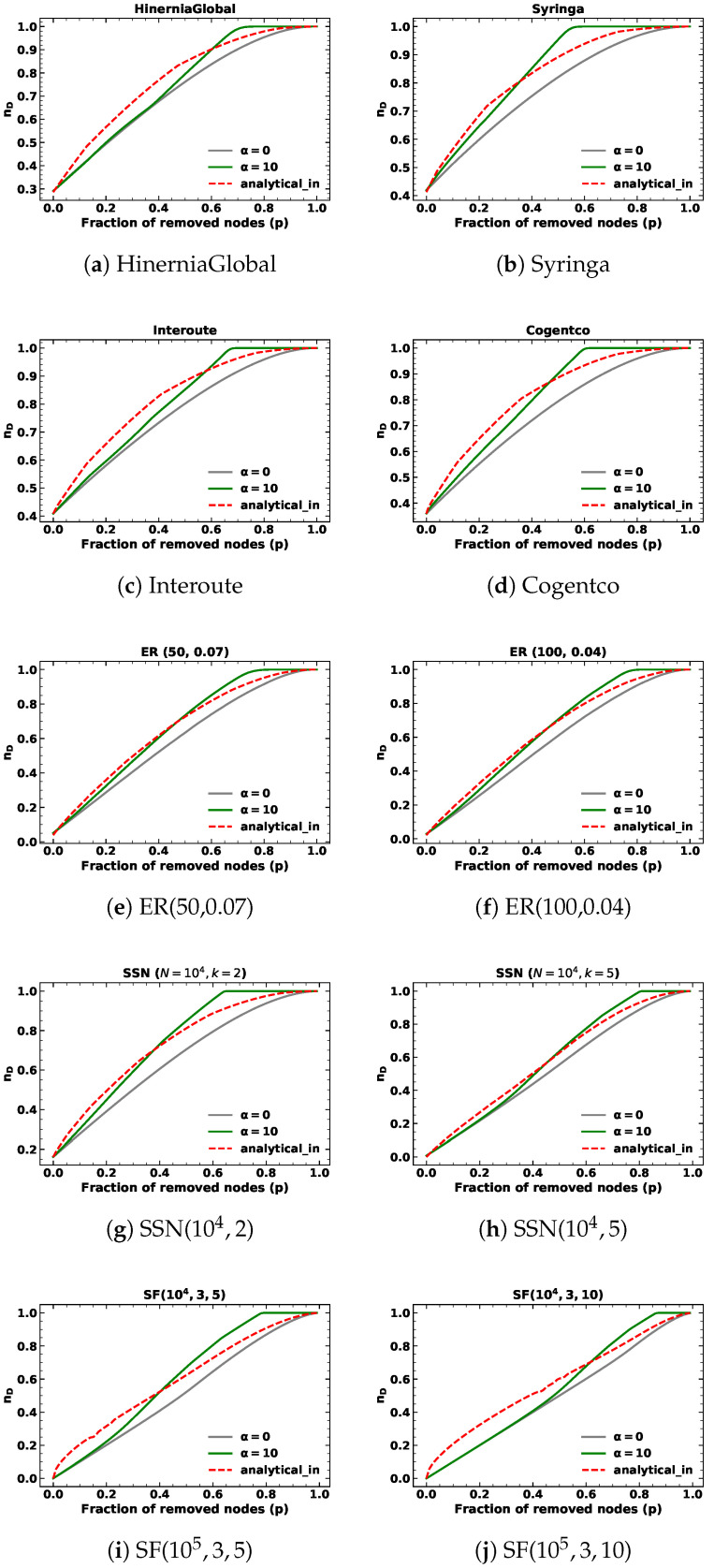
The minimum fraction of driver nodes $n_{D}$ during targeted node removal based on in-degree with α=10 for different kinds of networks.

**Figure 4:**
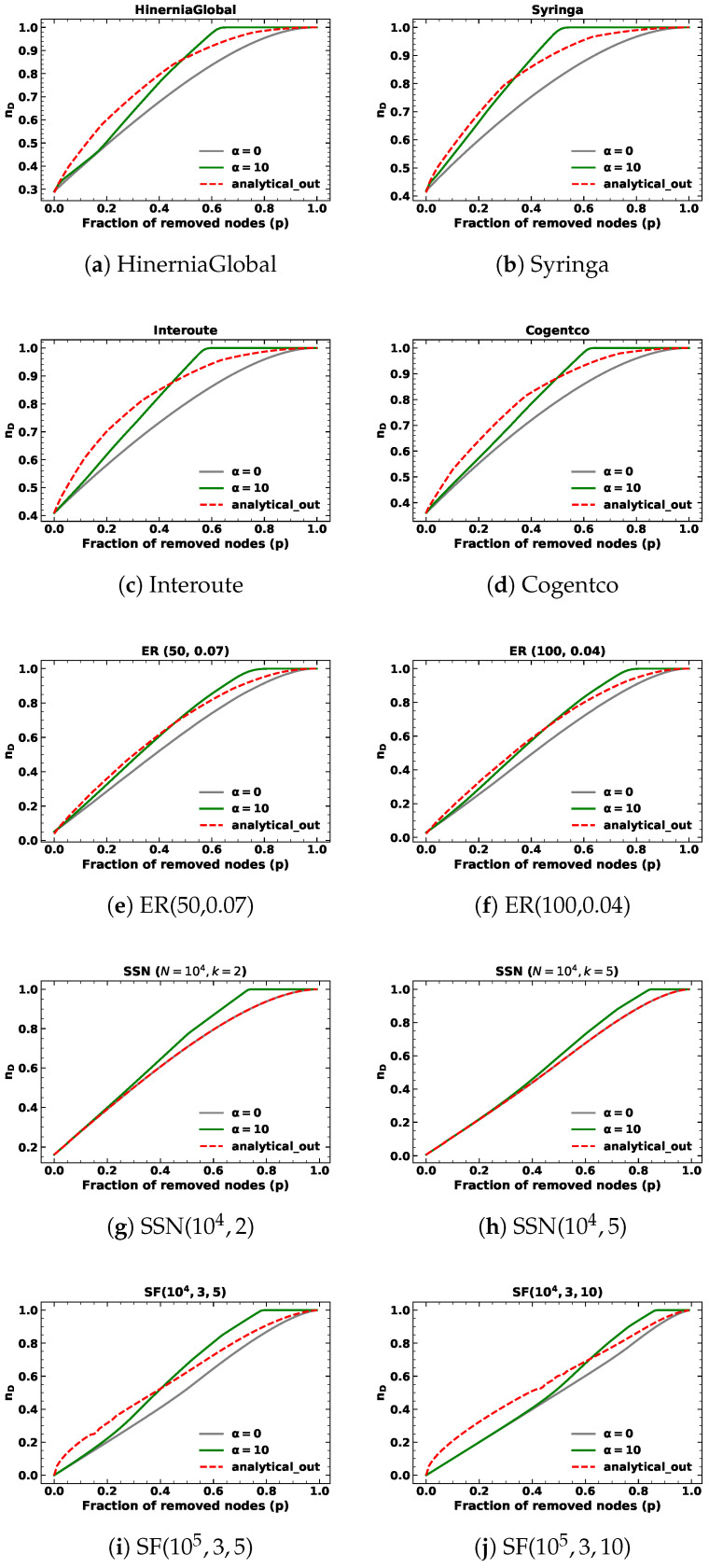
The minimum fraction of driver nodes $n_{D}$ during targeted node removal based on out-degree with α=10 for different kinds of networks.

We ran the simulations of 10,000 realizations with α=10 under in-degree and out-degree node removals in mentioned networks. Each realization of every network is the same as described in case α=1. The simulation results of network controllability are shown in the green lines in Figure 3 and Figure 4. As before, the analytical results are depicted in red lines, while the simulation results of network controllability under random node attacks are shown in gray lines.

In addition to targeted node removals based on out-degree in SSNs with fixed out-degree, the analytical results are consistent with random node removals. Notably, the analytical results exhibit a similar pattern for α=10, where they initially surpass the simulation results before eventually intersecting and becoming inferior to the targeted node attack lines but superior to the random node attack lines as the fraction of removed nodes approaches one. We find the proposed methods can closely approximate network controllability using a closed-form approach, but do not precisely align with simulation results.

Upon examining Table 2 and Table 3, we observe that both the proposed analytical methods and the analytical results of random node removal demonstrate satisfactory performance for targeted node removal based on in-degree and out-degree with α=10 when the fraction of removed nodes *p* is below 10%. However, the values obtained for α=10 are comparatively inferior to those obtained for α=1 and random node removal. These outcomes highlight the limitations of our proposed approach. Specifically, our method assumes that nodes are removed from the node with the highest degree to the node with the lowest degree, which is true when α is large enough, such as infinity. In this context, we choose α=10 and the node with the highest degree is much more likely to be removed, but still cannot be guaranteed to be removed at each step.

## 6. Conclusions and Discussion

This study introduces analytical methods based on generating functions to determine the minimum fraction of driver nodes required to maintain network controllability in directed networks under node failures based on in-degree and out-degree. We develop separate analytical techniques for two scenarios, namely α=1 and α=10. Our proposed analytical methods demonstrate reasonable results to predict the minimum fraction of driver nodes under targeted attacks. Furthermore, our investigation indicates that random node removal may also serve as a reliable predictor of the results of various targeted node removals, particularly when the fraction of removed nodes is minimal (below 10%).

In addition to the findings presented in this paper, we have endeavored to apply our simulations to various other real-world networks. Our analysis reveals that the minimum fraction of driver nodes calculated by the proposed analytical method utilizing generating functions does not coincide with the results obtained using the maximum matching algorithm before node removal. As such, our proposed methods are inadequate for predicting the minimum fraction of driver nodes under node removal for these networks. When targeted node removal is based on in-degree and out-degree with α=10, our approximation method assumes that nodes are removed in descending order of in-degree and out-degree. However, the assumption does not reflect the actual removal process, as we recalculated the removal probabilities to choose nodes at each step. This discrepancy is one of the reasons for the inaccurate results obtained. Moreover, we acknowledge that further improvements are required to enhance the method’s efficacy. Notably, the numerical solution of the predicted outcomes can be challenging to obtain, particularly when attempting to acquire the results for SFs with some other parameters.

The approximation of node removals based on in- or out-degree involves an assumption that the in-degree distribution and out-degree distribution evolve independently. However, the assumption requires further investigation to ensure its validity. To address this issue, an avenue of promising research involves examining the relationship between in-degree and out-degree distributions through the randomization of networks. Such analyses may provide upper and lower bounds for analytical methods, contributing to the improvement of predictions about network controllability under targeted attacks based on in-degree and out-degree.

In the future, we aim to broaden the scope of our findings by including other types of node attacks, specifically localized node attacks, as documented in [28]. Furthermore, we intend to verify our conclusions on a more comprehensive collection of real-world networks and various types of networks, such as interdependent networks. We also plan to apply additional prediction techniques, such as machine learning methods, to assess network controllability under node removals concerning in-degree and out-degree.

## Figures and Tables

**Table 1:** Properties of four real-world communication networks.

Name	*N*	*L*	<k>
HinerniaGlobal	55	81	2.95
Syringa	74	74	2.00
Interoute	110	146	2.65
Cogentco	197	243	2.47

**Table 2:** The RMSE between the analytical results of random removals and the simulation results under random removals, target removals with α=1 and α=10, respectively, while removing 10% of the nodes. The column labeled “Random” indicates the RMSE under random removals. The columns labeled “α=1” and “α=10” represent the RMSE under targeted node removals with α=1 and α=10, respectively. The columns labeled “Indegree”, “Outdegree”, and “Degree” represent the RMSE under targeted node removals based on in-degree, out-degree, and total degree, respectively. The analytical method for random removals is from the reference [19].

Network	Random	α=1			α=10		
		Indegree	Outdegree	Degree	Indegree	Outdegree	Degree
SF($10^{5}$, 3, 5)	0.0005	0.0010	0.0010	0.0010	0.0032	0.0032	0.0032
SF($10^{5}$, 3, 10)	0.0001	0.0001	0.0001	0.0001	0.0001	0.0001	0.0001
ER(50, 0.07)	0.0137	0.0164	0.0156	0.0155	0.0190	0.0195	0.0223
ER(100, 0.04)	0.0079	0.0086	0.0095	0.0094	0.0126	0.0121	0.0156
HinerniaGlobal	0.0039	0.0052	0.0110	0.0084	0.0025	0.0152	0.0152
Syringa	0.0071	0.0136	0.0217	0.0179	0.0237	0.0263	0.0443
Interoute	0.0011	0.0008	0.0106	0.0056	0.0064	0.0072	0.0175
Cogentco	0.0011	0.0090	0.0053	0.0071	0.0156	0.0091	0.0248
SSN($10^{4}$, 2)	0.0000	0.0103	0.0003	0.0052	0.0143	0.0007	0.0155
SSN($10^{4}$, 5)	0.0000	0.0006	0.0000	0.0003	0.0008	0.0001	0.0008

**Table 3:** The RMSE between the analytical results of the proposed analytical methods and the simulation results under different kinds of removals while removing 10% of the nodes. The column labeled “Random” indicates the RMSE under random removals. The columns labeled “α=1” and “α=10” represent the RMSE under targeted node removals with α=1 and α=10, respectively. The columns labeled “Indegree”, “Outdegree”, and “Degree” represent the RMSE under targeted node removals based on in-degree, out-degree, and total degree, respectively. The analytical methods for random removals and targeted node removals based on the total degree are from the reference [19].

Network	Random	α=1			α=10		
		Indegree	Outdegree	Degree	Indegree	Outdegree	Degree
SF($10^{5}$, 3, 5)	0.0005	0.0010	0.0010	0.0546	0.0764	0.0764	0.1573
SF($10^{5}$, 3, 10)	0.0001	0.0001	0.0001	0.0555	0.0799	0.0799	0.1600
ER(50, 0.07)	0.0137	0.0122	0.0113	0.0095	0.0588	0.0595	0.0543
ER(100, 0.04)	0.0079	0.0058	0.0067	0.0039	0.0189	0.0193	0.0284
HinerniaGlobal	0.0039	0.0089	0.0136	0.0025	0.0281	0.0349	0.0354
Syringa	0.0071	0.0096	0.0143	0.0061	0.0157	0.0235	0.0142
Interoute	0.0011	0.0151	0.0242	0.0009	0.0265	0.0454	0.0229
Cogentco	0.0011	0.0233	0.0244	0.0050	0.0314	0.0330	0.0322
SSN($10^{4}$, 2)	0.0000	0.0085	0.0002	0.0027	0.0331	0.0006	0.0343
SSN($10^{4}$, 5)	0.0000	0.0094	0.0000	0.0024	0.0167	0.0001	0.0264

**Figure A1:**
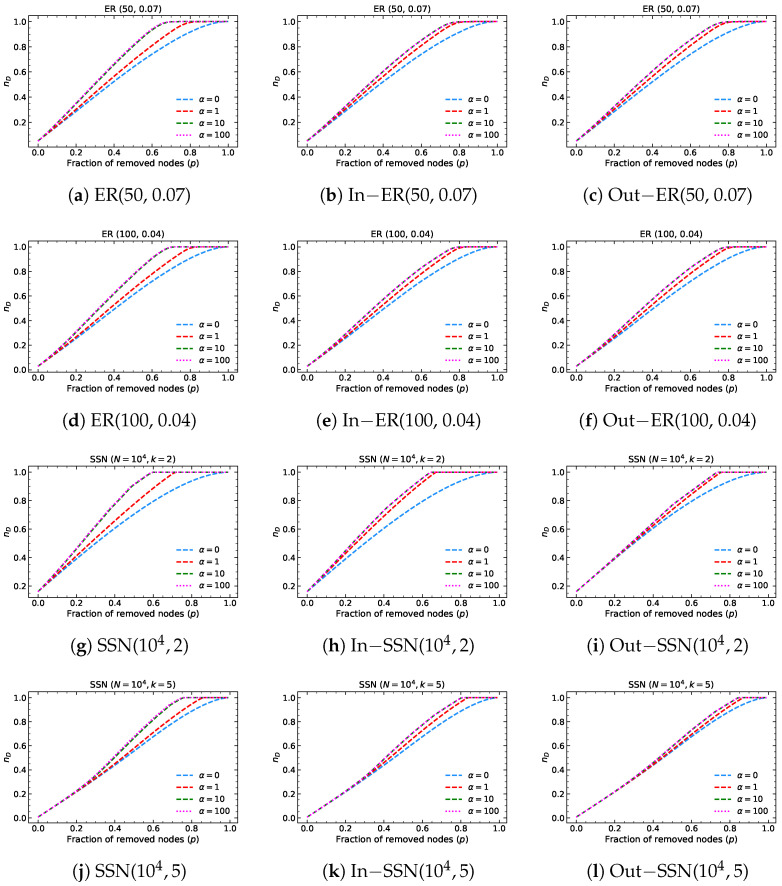
The minimum fraction of driver nodes $n_{D}$ during targeted node removal based on in-degree and out-degree with α=0, α=1, α=10 and α=100 for different kinds of networks. The results are the average $n_{D}$ calculated by the maximum matching algorithm over 10,000 realizations of real-world networks and 1000 realizations of model networks. The blue, orange, and green dashed lines are the results of simulations with α=0, α=1 and α=10 separately. The pink dotted lines are obtained by the simulation results with α=100.

**Figure A2:**
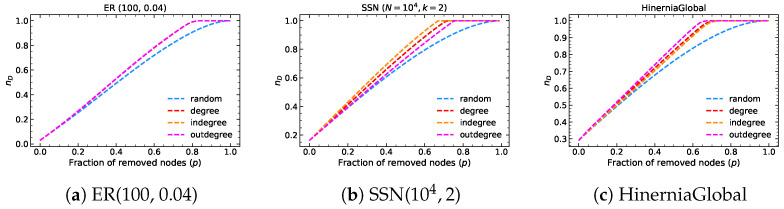
The minimum fraction of driver nodes $n_{D}$ during random removal and targeted node removal based on degree, in-degree and out-degree with α=1 for three networks. The results are the average $n_{D}$ calculated by the maximum matching algorithm over 10,000 realizations of HinerniaGlobal and 1000 realizations of ER(100, 0.04) and SSN($10^{4},2$). The blue, red, orange and pink dashed lines are the results of simulations with random removal, target removal based on the total degree with α=1, target removal based on in-degree with α=1 and target removal based on out-degree with α=1 separately.

**Figure A3:**
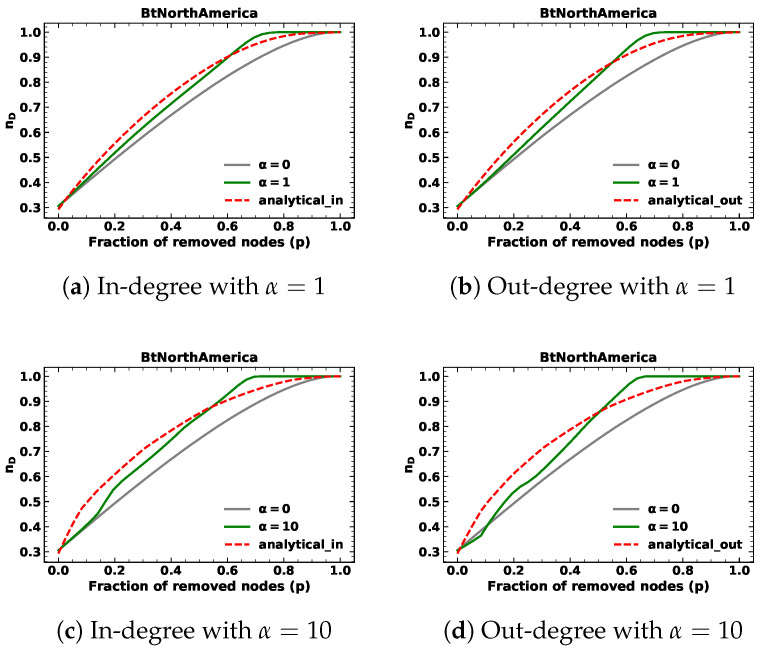
The minimum fraction of driver nodes $n_{D}$ during targeted node removal based on in-degree and out-degree with α=1 and α=10, respectively, for the network BtNorthAmerica.

## Data Availability

Not applicable.

## Abbreviations

The following abbreviations are used in this manuscript:

ER	Erdös-Rényi networks
SSNs	Swarm Signal networks
SFs	Scale-free networks
RMSE	Root Mean Square Error

## Appendix A. The Simulation Results Based on Different α Values

The following figures demonstrate for α=0, α=1, α=10 and α=100 how the minimum fraction of driver nodes changes under targeted attacks based on degree, in-degree and out-degree for ER networks and SSNs. We find that the results of α=10 and α=100 overlap.

## Appendix B. Comparison with Node Removal Based on Degree with α=1

We present the results of four types of node removal strategies: random removal, targeted node removal based on the total degree with α=1, targeted node removal based on in-degree with α=1, and targeted node removal based on out-degree with α=1 for three networks in Figure A2. We find that the three targeted node removal strategies are more disruptive than random removal. However, the effectiveness of the targeted node removal strategies varies depending on the network structure. For instance, in ER(100,0.04), all three targeted node removal strategies show similar performance. In SSN($10^{4},2$), the targeted node removal based on in-degree is the most disruptive; whereas, in HinerniaGlobal, the targeted node removal based on out-degree is the most disruptive.

## Appendix C. Another Real-World Network Results

In this study, the real-world graphs utilized have an average degree ranging from 2 to 3. To further evaluate the efficacy of the proposed techniques, we selected a network from the Topology Zoo dataset, namely BtNorthAmerica, which possesses an average total degree of 4.22. The network under consideration comprises 36 nodes and 76 links. We analyzed the controllability of the network under node removals concerning node in-degree and out-degree with different α. The results are presented in Figure A3 and Table A1 and Table A2. Our findings suggest that the predicted values of the proposed methods are valid. It is worth mentioning that, when α=10, attacks based on out-degree at the onset are not as deleterious as random removals. Removing the node with the highest out-degree in the initial steps results in a lower average number of driver nodes than removing other nodes, on average.

**Table A1:** The RMSE between the analytical results of random removals and the simulation results under random removals, target removals with α=1 and α=10, respectively, while removing 10% of the nodes. The analytical method for random removals is from the reference [19].

Network	Random	α=1			α=10		
		Indegree	Outdegree	Degree	Indegree	Outdegree	Degree
BtNorthAmerica	0.0097	0.0140	0.0104	0.0121	0.0117	0.0096	0.0101

**Table A2:** The RMSE between the analytical results of the proposed analytical methods and the simulation results under different kinds of removals while removing 10% of the nodes. The analytical methods for random removals and targeted node removals based on the total degree are from the reference [19].

Network	Random	α=1			α=10		
		Indegree	Outdegree	Degree	Indegree	Outdegree	Degree
BtNorthAmerica	0.0097	0.0126	0.0175	0.0091	0.0538	0.0612	0.0527

## Appendix D. Analytical Approximation of Random Node Removals about SFs

This section shows the analytical solution for random node removal in SFs. In SFs, the in-degree distribution and out-degree distribution both follow the pure power-law distribution with minimum degree *a* and exponent γ, which can be denoted as follows:

(A1) $$\begin{array}{c} P_{in}\left(k_{in}\right)=C_{in}k_{in}^{-\gamma },\;P_{out}\left(k_{out}\right)=C_{out}k_{out}^{-\gamma }, \end{array}$$

where $C_{in}=\frac{1}{\sum_{k_{in}=a}^{\infty }k_{in}^{-\gamma }}=\frac{1}{\zeta (\gamma ,a)}$ and $C_{out}=\frac{1}{\sum_{k_{out}=a}^{\infty }k_{out}^{-\gamma }}=\frac{1}{\zeta (\gamma ,a)}$, where ζ(γ,a) is the Hurwitz Zeta function, and the average degree $k=\frac{\zeta (\gamma -1,a)}{\zeta (\gamma ,a)}$. Correspondingly, the generating functions can be obtained by

(A2) $$\begin{array}{c} G_{in}\left(x\right)=\frac{x^{a}\Phi \left(x,\gamma ,a\right)}{\zeta (\gamma ,a)},\;G_{out}\left(x\right)=\frac{x^{a}\Phi \left(x,\gamma ,a\right)}{\zeta (\gamma ,a)}, \end{array}$$

where Φ(z,s,α) denotes the Lerch transcendent function.

Together with Equations (9) and (10), the fraction of the minimum number of driver nodes $n_{D}$ after randomly removing a fraction *p* nodes can be calculated by

(A3) $$\begin{array}{cc} n_{D} & =\frac{\left(1-p\right){\left(-p\omega_{2}+p+\omega_{2}\right)}^{a}\Phi \left(-p\omega_{2}+p+\omega_{2},\gamma ,a\right)}{\zeta (\gamma ,a)}+\\ \; & \frac{\left(1-p\right)\Phi \left(\frac{\left(p-1\right)\Phi \left(-p\omega_{2}+p+\omega_{2},\gamma -1,a\right){\left(-p\omega_{2}+p+\omega_{2}\right)}^{a-1}}{\zeta (\gamma -1,a)}+1,\gamma ,a\right)}{\zeta (\gamma ,a)}\\ \; & \times {\left(\frac{\left(p-1\right){\left(-p\omega_{2}+p+\omega_{2}\right)}^{a-1}\Phi \left(-p\omega_{2}+p+\omega_{2},\gamma -1,a\right)}{\zeta (\gamma -1,a)}+1\right)}^{a}\\ \; & +\frac{k\left(2p-1-p^{2}\right)\left(\omega_{2}-1\right){\left(-p\omega_{2}+p+\omega_{2}\right)}^{a-1}\Phi \left(-p\omega_{2}+p+\omega_{2},\gamma -1,a\right)}{\zeta (\gamma -1,a)}+2p-1 \end{array}$$

where $1-\omega_{2}-{\bar{H}}_{out}\left(1-{\bar{H}}_{in}\left(\omega_{2}\right)\right)=0$.
